# Umbilical cord blood miR-125b-5p: a diagnostic and prognostic biomarker for neonatal respiratory distress syndrome

**DOI:** 10.1590/1984-0462/2026/44/2025344

**Published:** 2026-10-19

**Authors:** Yang Zhang, Zhiqiang Liu, Yanchun Zhang, Yanan Hou, Lushuang Sun

**Affiliations:** a Shengli Oilfield Central Hospital, Shandong, China.

**Keywords:** Neonatal respiratory distress syndrome, Umbilical cord blood, miR-125b-5p, Diagnosis, Prognosis, Newborn, Síndrome do desconforto respiratório do recém-nascido, Sangue de cordão umbilical, miR-125b-5p, Diagnóstico, Prognóstico, Recém-nascido

## Abstract

**Objective:**

Neonatal respiratory distress syndrome (NRDS) is a common respiratory disease. This study aimed to detect the expression of miR-125b-5p in the umbilical cord blood of NRDS patients and to evaluate its diagnostic and prognostic value.

**Methods:**

Fluorescence-based real-time quantitative polymerase chain reaction was used to detect miR-125b-5p expression in the umbilical cord blood of NRDS patients and controls. The correlation between miR-125b-5p expression and NRDS clinical data was analyzed. Survival was analyzed using the Kaplan-Meier method. Cox regression identified risk factors for adverse outcomes, and the ROC curve evaluated diagnostic and prognostic accuracy.

**Results:**

The level of miR-125b-5p in NRDS patients was higher than that in controls. High miR-125b-5p expression was correlated with C-reactive protein levels, umbilical artery blood gas parameters, and the lung ultrasound score. Patients with high miR-125b-5p expression had significantly shorter event-free survival (HR 2.358, 95%CI 1.345-4.133, p<0.05) and long-term survival (HR 2.489, 95%CI 1.201-5.161, p<0.05). Cox regression identified high miR-125b-5p expression, high lung ultrasound score, high PaCO₂, and high PaO₂ as prognostic factors.

**Conclusions:**

Umbilical cord blood miR-125b-5p is highly expressed in NRDS. It may serve as a biomarker for the diagnosis and prognosis of NRDS.

## RESUMO

**Objetivo:**

A síndrome do desconforto respiratório (SDR) do recém-nascido é uma doença respiratória comum. O presente estudo teve como objetivo testar a expressão de miR-125b-5p no sangue do cordão umbilical de pacientes com SDR e avaliar sua eficácia no diagnóstico e prognóstico desses pacientes.

**Métodos:**

A reação em cadeia da polimerase quantitativa por fluorescência em tempo real foi usada para detectar a expressão de miR-125b-5p no sangue do cordão umbilical de recém-nascidos e controles com SDR. A correlação entre a expressão de miR-125b-5p e os dados clínicos da SDR foi analisada. A sobrevida foi analisada pela curva de Kaplan-Meier. A regressão de Cox identificou os fatores de risco para desfechos adversos, e a curva ROC avaliou a acurácia diagnóstica e prognóstica.

**Resultados:**

O nível de miR-125b-5p em recém-nascidos com SDR foi maior do que nos controles. A expressão elevada de miR-125b-5p correlacionou-se com os níveis de proteína C-reativa, os parâmetros gasométricos da artéria umbilical e o escore ultrassonográfico pulmonar. Pacientes com alta expressão de miR-125b-5p tiveram sobrevida livre de eventos significativamente menor (HR 2,358, IC95% 1,345-4,133, p<0,05) e sobrevida livre de progressão (HR 2,489, IC95% 1,201-5,161, p<0,05). A regressão de Cox identificou que a alta expressão de miR-125b-5p, alto escore ultrassonográfico pulmonar, alta PaCO₂ e alta PaO₂ foram os fatores prognósticos.

**Conclusões:**

O miR-125b-5p no sangue do cordão umbilical é altamente expresso na SDR, podendo servir como biomarcador para o diagnóstico e prognóstico da SDR.

## INTRODUCTION

Neonatal respiratory distress syndrome (NRDS) is a severe respiratory disorder, but it is different from acute respiratory distress syndrome (ARDS).^1^ NRDS is a disease primarily caused by a deficiency of pulmonary surfactant.^2^ The clinical features mainly include shortness of breath, wheezing, the three-depression sign during inhalation, and nasal flaring.^3^ Severe cases of NRDS may even progress to respiratory failure, threatening patients’ lives.^4^ Therefore, early detection and treatment of NRDS are crucial to patient survival.

Currently, the diagnosis of NRDS in clinical practice mainly relies on clinical manifestations, blood gas analysis, and lung ultrasound (LUS).^5^ With increasing understanding of the pathogenesis of NRDS, molecular diagnostic techniques have gradually become an important approach for the early identification and precise treatment of NRDS.^6^ ^,^ ^7^ Among these techniques, microarray analysis - as a high-throughput molecular biology method - can simultaneously detect the expression levels of thousands of genes or molecules, thereby achieving rapid screening and quantitative analysis of disease-related biomarkers.^8^ ^,^ ^9^ ^,^ ^10^

MicroRNAs (miRNAs) have become a key tool in molecular diagnosis.^11^ At present, the role of miRNAs in the diagnosis and prognosis of respiratory distress is constantly being recognized.^12^ ^,^ ^13^ Studies have shown that upregulation of miR-375 in umbilical cord blood is closely associated with the risk of NRDS and its clinical prognosis.^14^ Another study indicated that circulating miR-363 expression is significantly decreased in NRDS patients compared with healthy controls.^15^ Furthermore, a study on placental miRNAs revealed that the expression of miR-125b-5p in umbilical cord plasma was altered in NRDS patients.^16^ However, the value of miR-125b-5p in NRDS remains to be explored.

Accordingly, the expression level of miR-125b-5p was measured in the umbilical cord blood of neonates with NRDS. The potential application value of miR-125b-5p level was investigated in NRDS patients, along with arterial blood gas parameters and LUS score, in the prognosis assessment of NRDS.

## METHOD

This study was a single-center retrospective cohort study conducted at Shengli Oilfield Central Hospital. The eligible population comprised 348 premature patients admitted to our hospital between January 2022 and January 2024. After screening against the inclusion and exclusion criteria, 159 patients were diagnosed with NRDS, whereas 189 patients were not. To balance baseline characteristics and reduce confounding bias, we selected 60 patients without NRDS or other severe complications from the 189 non-NRDS patients. These participants were matched to the NRDS patients in terms of gestational age and birth weight and served as the control.

The inclusion criteria for the NRDS patients were:

1. Premature infants with a gestational age at birth of less than 37 weeks;
2. Diagnosed with NRDS based on clinical symptoms, chest imaging findings, and typical clinical course.^17^
3. Umbilical cord blood samples collected within 24 hours after birth;
4. Complete clinical and follow-up data available.

The exclusion criteria were:

1. Presence of congenital heart disease, genetic metabolic diseases, or severe congenital malformations;
2. Maternal use of glucocorticoids for more than three courses during pregnancy;
3. Insufficient umbilical cord blood samples or hemolyzed samples;
4. Substantial lack of clinical data or follow-up information.

According to the Montreux diagnostic criteria and the oxygenation index (OI), the patients were divided into non-critical group (OI 4≤OI<8; n=70), critical group (8≤OI<16; n=53), and extremely critical group (OI≥16, n=36).^18^ This research was approved by the Shengli Oilfield Central Hospital Ethics Committee (approval No.: YXLL202100701, date: April 11, 2021). Written informed consent was obtained from all patients’ guardians before study enrollment.

General information of all patients was collected, including gestational age, birth weight, gender, delivery mode, intrauterine distress, and maternal gestational hypertension. In addition, the C-reactive protein (CRP) level in umbilical cord blood was measured using the immunoturbidimetric method.

Total RNA was extracted from umbilical cord blood serum using TRIzol reagent (Invitrogen, USA). The purity and concentration of RNA were determined using a NanoDrop 2000 (Thermo Fisher Scientific, USA). Reverse transcription of miR-125b-5p was performed using the ABI 7500 fluorescence-based real-time quantitative polymerase chain reaction (RT-qPCR) instrument (Applied Biosystems, USA) using SYBR Green I Master Mix kit (Invitrogen, USA). U6 small nuclear RNA (U6 snRNA) was used as the internal reference gene for miR-125b-5p, and the primers were synthesized by Shanghai Sangon Biotech Co., Ltd. The primer sequences were as follows: miR-125b-5p forward, 5’-GCTCCCTGAGACCCTAAC-3’; reverse, 5’-GTGCGTGTCGTGGAGTCG-3’; U6 snRNA forward, 5’-TAATCTCAGCTGGCAACTGTGA-3’; and reverse, 5’-TCACAGTTGCCAGCTGAGATTA-3’. The relative expression of miR-125b-5p was calculated using the 2^-^ΔΔ^Ct^ method. The specific calculation formula was: ΔCt=Ct (target gene) - Ct (reference gene), ΔΔCt=ΔCt (experimental group) - ΔCt (control group), and the relative expression level of the target gene=2^-^ΔΔ^Ct^. Each sample had three replicates, and the average value was used for statistical analysis.

According to the American College of Obstetricians and Gynecologists (ACOG) consensus and obstetric guidelines, all patients uniformly underwent delayed umbilical cord ligation. The umbilical cord was ligated 30-60 seconds after the fetus was delivered, using two hemostatic forceps to clamp the proximal end of the umbilical cord to block the blood circulation between the newborn and the placenta.^19^ Under sterile conditions, a syringe pre-filled with heparin was used to collect umbilical artery blood. Of this sample, 2 mL of umbilical artery blood was used for blood gas analysis. After sampling was completed, the tip of the syringe needle was immediately sealed with a rubber stopper to prevent air exposure or contamination. The collected umbilical artery blood sample was sent to the laboratory for testing within 30 minutes after collection. A fully automatic blood gas analyzer (Werfen, USA) was used to measure blood gas indicators in the umbilical artery. The parameters analyzed included potential of hydrogen (pH), partial pressure of oxygen (PaO₂), partial pressure of carbon dioxide (PaCO₂), and base excess (BE). Another 2 mL of umbilical artery blood was collected, and the level of CRP in the umbilical cord blood was determined using the immunoturbidimetric method. The remaining 5 mL of umbilical artery blood was aliquoted and stored at -80^o^C in an ultra-low temperature refrigerator to prevent repeated freezing and thawing of the samples. Subsequently, total RNA extraction and RT-qPCR analysis were performed to quantify the expression level of miR-125b-5p.

LUS examinations were performed by two physicians with over five years of experience in neonatal ultrasound. A color Doppler ultrasound diagnostic instrument (Siemens AG, Germany, #AU800) with a probe frequency of 9-15 MHz was used. Before each inspection, the equipment and probe were calibrated according to the original standard procedure. The patients were placed in the supine position. The lungs were divided into 12 regions using the sternal midline, axillary anterior line, axillary posterior line, posterior midline, and bilateral nipple lines. The scoring method was based on previous studies.^20^ ^,^ ^21^ The score range for each region was 0-36 points. The LUS score was the total score of the 12 regions. A higher score indicated a more severe condition. The final classification was based on the consensus of the two physicians’ diagnoses. Thirty percent of the cases were randomly selected for re-measurement. If the intragroup correlation coefficient was greater than 0.75, it was considered to indicate good consistency.

Long-term follow-up of the patients was conducted until the corrected gestational age of 12 months. Event-free survival was defined as the time from study enrollment to the first occurrence of severe complications such as bronchopulmonary dysplasia, intracranial hemorrhage, sepsis, patent ductus arteriosus, pulmonary hemorrhage, neurological abnormalities, or all-cause death. The primary outcome measure of this study was the neurodevelopmental status evaluated using the Gesell Developmental Scale at the corrected gestational age of 12 months. The secondary outcome was the first occurrence of the aforementioned severe complications during the follow-up period. Poor prognosis was defined as death, discontinuation of follow-up due to severe complications, or occurrence of any severe complication during follow-up. A good prognosis referred to survival through the entire follow-up period without severe complications and the completion of the final assessment. Any death or irreversible severe complication during follow-up was also recorded as a terminal event.

Statistical analysis and graph generation were conducted using Statistical Package for the Social Science (SPSS) version 26.0 and GraphPad Prism 9.0 software. Data normality was assessed by the Shapiro-Wilk test. Data were expressed as mean ± standard deviation (x±s). For continuous variables, an independent-samples *t*-test was used for intergroup comparison; for categorical variables, the ꭓ^2^ test was employed. The Pearson correlation coefficient was used to investigate the correlation between the expression of miR-125b-5p and LUS scores. Based on the median expression of miR-125b-5p in cord blood, the NRDS patients were divided into the miR-125b-5p high-expression group (n=79) and the miR-125b-5p low-expression group (n=80). The Kaplan-Meier survival curve analysis was used to investigate the association between the expression level of miR-125b-5p and event-free survival, as well as long-term survival in NRDS patients, and the log-rank test was used to compare survival between groups. Continuous variables such as gestational age, birth weight, miR-125b-5p level, blood gas parameters, and LUS score were directly included in the Cox regression analysis in their original forms; binary variables such as gender (male/female), mode of delivery (vaginal delivery/cesarean section), intrauterine distress (present/absent), and maternal pregnancy-induced hypertension (present/absent) were included in the Cox regression analysis in the form of 0/1 dummy variables. The proportional hazards assumption was verified through the Schoenfeld residual test, and all variables met the prerequisite conditions for proportional hazards (p>0.05). Variables with a variance inflation factor (VIF)<3 were considered to have no significant multicollinearity. Receiver operating characteristic (ROC) curves were used to evaluate the predictive performance. The optimal cutoff value of the ROC curve was determined by the maximum Youden index (Youden index=sensitivity+specificity-1). The sample size for the ROC curve analysis was calculated using MedCalc statistical software, with parameters set as an expected area under the curve (AUC)=0.80, two-sided α=0.05, and power (1-β)=0.90 based on the Hanley and McNeil method.^22^ The results showed that at least 58 patients weree required in each group. This study ultimately included 60 controls and 159 NRDS patients, meeting and exceeding the minimum sample size requirement. A p<0.05 was considered statistically significant.

## RESULTS

Baseline clinical data were compared between the control group and NRDS patients were compared, and the results are shown in Table 1. There was no statistically significant difference between the two groups of patients in terms of gestational age, gender, birth weight, delivery mode, intrauterine distress, and maternal gestational hypertension (p>0.05). The RT-qPCR results showed that the expression of miR-125b-5p in NRDS patients was significantly higher than that in the control group (p*<*0.001; Figure 1A). Further ROC curve analysis revealed that the AUC of miR-125b-5p was 0.749. When the cutoff value was set at 1.395, the sensitivity was 55.97%, and the specificity was 85.00% (Figure 1B).

**Table 1.** Comparison of baseline clinical characteristics between preterm infants with neonatal respiratory distress syndrome and healthy preterm controls.

Variables	Control	NRDS	p-value
Gestational age (weeks)	30.29±2.38	30.88±2.67	0.131
Birth weight (g)	1885.35±337.45	1868.08±280.06	0.701
Gender, n (%)			
Male	31 (51.67)	77 (48.43)	0.669
Female	29 (48.33)	82 (51.57)	
Delivery mode, n (%)			
Vaginal	44 (73.33)	129 (81.13)	0.206
Cesarean section	16 (26.67)	30 (18.87)	
Intrauterine distress, n (%)			
Yes	18 (30.00)	32 (20.13)	0.121
No	42 (70.00)	127 (79.87)	
Maternal gestational hypertension, n (%)			
Yes	12 (20.00)	24 (15.09)	0.382
No	48 (80.00)	135 (84.91)	

NRDS: neonatal respiratory distress syndrome.

Continuous variables (gestational age, birth weight) are presented as mean±standard deviation and analyzed using and independent samples *t*-test. Categorical variables are expressed as n (%) and compared using the ꭓ^2^ test.

Source: The authors.

**Figure 1.**
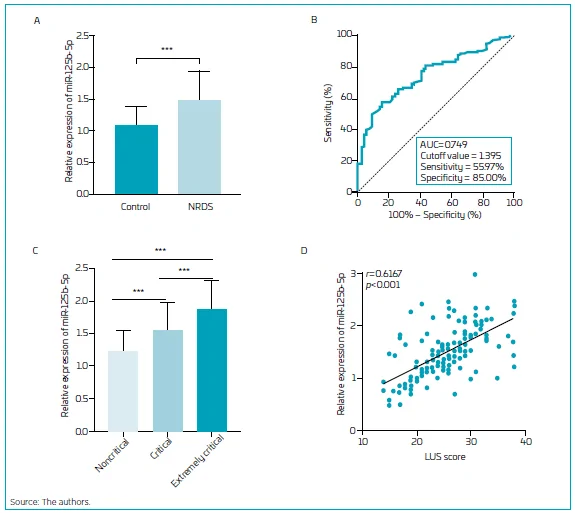
This study included a total of 159 patients with NRDS and 60 healthy controls. (A) Comparison of the relative expression of miR-125b-5p between the NRDS group and the control group. Intergroup differences were assessed using independent samples t-test, p<0.001. (B) Receiver operating characteristic (ROC) curve analysis for the diagnosis of NRDS. The AUC was 0.749, the cutoff value was 1.395, the sensitivity was 55.97%, and the specificity was 85.00%. (C) Relative expression of miR-125b-5p among the non-critical (n=70), critical (n=53), and extremely critical groups (n=36). One-way analysis of variance (ANOVA) followed by post-hoc Tukey test was adopted for multi-group comparison, p<0.001. (D) Correlation analysis of miR-125b-5p expression with the lung ultrasound (LUS) score. r=0.6167, p<0.001. Statistical significance markers: ***p<0.001. Relative expression of miR-125b-5p was calculated using the 2-ΔΔCt method and normalized to U6 small nuclear RNA (snRNA). The expression level in the control group was set to 1, and values represent fold changes relative to the control.

In this study, NRDS patients were classified into non-critical group (n=70), critical group (n=53), and extremely critical group (n=36) based on the severity of their condition. The results showed that as the severity of NRDS increased, the level of miR-125b-5p in the umbilical cord blood showed a significant upward trend (all p*<*0.001; Figure 1C). Correlation analysis revealed that the expression of miR-125b-5p in the umbilical cord blood was significantly positively correlated with the LUS score (r=0.6167, p<0.001; Figure 1D).

There were no statistically significant differences in gestational age, birth weight, gender, delivery mode, intrauterine distress, and maternal gestational hypertension between the miR-125b-5p low-expression group and the high-expression group. However, CRP, blood gas parameters, and LUS score showed significant differences between the two groups (all p<0.001; Table 2).

**Table 2.** Correlation analysis between miR-125b-5p expression levels and clinical parameters in neonatal respiratory distress syndrome patients.

Variables	miR-125b-5p expression		p-value
	Low (n=80)	High (n=79)	
Gestational age (weeks)	30.81±2.82	30.95±2.52	0.736
Birth weight (g)	1853.36±284.78	1882.98±276.22	0.507
Gender, n (%)			
Male	41 (51.25)	36 (45.57)	0.474
Female	39 (48.75)	43 (54.43)	
Delivery mode, n (%)			
Vaginal	12 (15.00)	18 (22.78)	0.210
Cesarean section	68 (85.00)	61 (77.22)	
Intrauterine distress, n (%)			
Yes	14 (17.50)	18 (22.78)	0.406
No	66 (82.50)	61 (77.22)	
Maternal gestational hypertension, n (%)			
Yes	10 (12.50)	14 (17.72)	0.358
No	70 (87.50)	65 (82.28)	
pH	7.26±0.08	7.16±0.07	<0.001
PaCO_2_ (mmHg)	53.25±3.75	57.28±2.52	<0.001
PaO_2_ (mmHg)	59.46±7.36	50.02±6.63	<0.001
BE (mmol/L)	-7.91±1.39	-8.66±1.51	<0.001
CRP (mg/L)	25.58±3.68	29.57±2.63	<0.001
LUS score	22.89±4.20	28.22±5.54	<0.001

pH: potential of hydrogen; PaCO_2_: arterial carbon dioxide partial pressure; PaO_2_: arterial oxygen partial pressure; BE: base excess; CRP: C-reactive protein; LUS: lung ultrasound.

Relative expression of miR-125b-5p was calculated using the 2^^-^ΔΔ^Ct^ method and normalized to U6 snRNA. The expression level in the control group was set TO 1, and values represent fold changes relative to the control. All data are presented as mean±standard deviation or n (%).

Source: The authors.

Results showed that NRDS patients with high miR-125b-5p expression had significantly shorter event-free survival (HR 2.358; 95%CI 1.345-4.133; p<0.05; Figure 2A) and long-term survival (HR 2.489; 95%CI 1.201-5.161; p<0.05; Figure 2B) compared with those with low miR-125b-5p expression. Further analysis revealed that the expression of miR-125b-5p was significantly higher in NRDS patients with poor prognosis than in those with good prognosis (p<0.001; Figure 2C).

**Figure 2.**
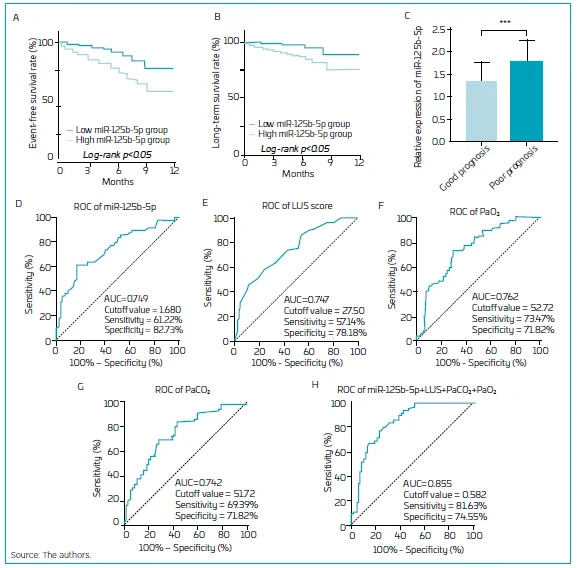
All 159 NRDS patients were stratified into miR-125b-5p low-expression group (n=80) and high-expression group (n=79) based on the median expression value of miR-125b-5p. (A) Kaplan-Meier curves for event-free survival comparison between miR-125b-5p high- and low-expression groups. Log-rank p<0.05, HR=2.358 (95%CI 1.3454.133). (B) Kaplan-Meier curves for long-term overall survival comparison between miR-125b-5p high- and low-expression groups. Log-rank p<0.05, HR=2.489 (95%CI 1.2015.161). (C) Relative expression of miR-125b-5p in NRDS patients with poor or good prognosis during follow-up. Intergroup differences were assessed using independent samples t-test, p<0.001. (D) Receiver operating characteristic (ROC) curve of miR-125b-5p. The AUC was 0.749, the cutoff value was 1.680, the sensitivity was 61.22%, and the specificity was 82.73%. (E) ROC curve of lung ultrasound (LUS) score. The AUC was 0.747, the cutoff value was 27.5, the sensitivity was 57.14%, and the specificity was 78.18%. (F) ROC curve of partial pressure of oxygen (PaO_2_). The AUC was 0.762, the cutoff value was 52.72, the sensitivity was 73.47%, and the specificity was 71.82%. (G) ROC curve of partial pressure of carbon dioxide (PaCO_2_). The AUC was 0.742, the cutoff value was 51.72, the sensitivity was 69.39%, and the specificity was 71.82%. (H) ROC curve of the combined model including miR-125b-5p, combined with LUS score, PaCO_2_, and PaO_2_. The AUC was 0.855, the cutoff value was 0.582, the sensitivity was 81.63%, and the specificity was 74.55%. Statistical significance was defined as p<0.05.

The results of the Cox regression analysis are shown in Table 3. The univariate regression analysis showed that high expression of miR-125b-5p (p=0.001), pH value (p=0.043), PaCO_2_ (p<0.001), PaO_2_ (p=0.002), BE (p=0.018), CRP (p=0.002), and LUS score (p<0.001) were significantly associated with prognosis, while gestational age, birth weight, gender, delivery mode, intrauterine distress, and maternal gestational hypertension were not significantly associated with prognosis. The indicators with p<0.05 in the univariate regression analysis were included in the multivariate analysis. The results showed that high expression of miR-125b-5p, high PaCO_2_, high LUS score, and high PaO_2_ were independent prognostic factors.

**Table 3.** Univariate and multivariate Cox regression analyses for predictors of poor prognosis in neonatal respiratory distress syndrome patients.

Variables	Univariate analysis			Multivariate analysis		
	HR	95%CI	p-value	HR	95%CI	p-value
Gestational age	0.737	0.415-1.310	0.298	-	-	-
Birth weight	0.792	0.449-1.397	0.421	-	-	-
Gender	0.576	0.320-1.038	0.066	-	-	-
Delivery mode	1.664	0.936-2.958	0.083	-	-	-
Intrauterine distress	1.263	0.719-2.219	0.416	-	-	-
Maternal gestational hypertension	1.227	0.700-2.152	0.475	-	-	-
pH	0.553	0.312-0.981	0.043	0.776	0.419-1.436	0.419
PaCO_2_	9.175	3.285-25.626	<0.001	8.558	2.349-31.177	0.001
PaO_2_	0.335	0.167-0.673	0.002	0.360	0.142-0.922	0.033
BE	0.462	0.244-0.874	0.018	0.775	0.393-1.528	0.462
CRP	2.759	1.462-5.208	0.002	1.563	0.767-3.187	0.219
LUS score	6.889	3.089-15.367	<0.001	4.738	1.884-11.918	<0.001
miR-125b-5p	2.842	1.507-5.359	0.001	1.978	1.020-3.835	0.043

HR: hazard ratio; CI: confidence interval; pH: potential of hydrogen; PaCO_2_: arterial carbon dioxide partial pressure; PaO_2_: arterial oxygen partial pressure; BE: base excess; CRP: C-reactive protein; LUS: lung ultrasound.

Source: The authors.

The ROC curve results showed that the AUC of miR-125b-5p was 0.749, the cutoff value was 1680, the sensitivity was 61.22%, and the specificity was 82.73% (Figure 2D); the AUC of LUS score was 0.747, the cutoff value was 27.50, the sensitivity was 57.14%, and the specificity was 78.18% (Figure 2E); the AUC of PaO_2_ was 0.762, the cutoff value was 52.72, the sensitivity was 73.47%, and the specificity was 71.82% (Figure 2F); the AUC of PaCO_2_ was 0.742, the cutoff value was 51.72, the sensitivity was 69.39%, and the specificity was 71.82% (Figure 2G). After further combined analysis of the above indicators, the AUC increased to 0.855, the cutoff value was 0.582, the sensitivity was 81.63%, and the specificity was 74.55% (Figure 2H).

### DISCUSSION

NRDS is a significant cause of neonatal respiratory failure^23^. Exploring effective diagnostic and prognostic markers is crucial for identifying the risk of poor prognosis in NRDS patients. This study found that the abnormal expression of miR-125b-5p in umbilical cord blood of NRDS patients was correlated with arterial blood gas parameters and the LUS score and could predict the clinical prognosis of NRDS. These findings enrich the research on molecular markers of NRDS and provide a reliable theoretical basis for formulating prognostic assessment strategies.

miRNAs can be stably detected in umbilical cord blood. The stably expressed miRNAs in umbilical cord blood have significant application value in the diagnostic and prognostic assessment of NRDS.^24^ ^,^ ^25^ Gusar et al. conducted RT-PCR on the umbilical cord blood of NRDS patients and found that the expression of miR-125b-5p in umbilical cord plasma showed significant changes.^16^ This is consistent with the findings of the present study. The experiment found that the level of miR-125b-5p in NRDS patients was higher than that in controls. In addition, a ROC curve analysis of the expression level of miR-125b-5p was performed. The results indicated that miR-125b-5p might serve as a potential biomarker for the diagnosis of NRDS. This result not only further enriches the research evidence on miRNAs related to NRDS but also provides a new target reference for the subsequent development of miRNA-based diagnostic and prognostic assessment tools for NRDS.

The LUS score is a core indicator for evaluating the condition of NRDS.^26^ The single LUS score has certain limitations in the assessment of NRDS. Existing studies have shown that the combined expression levels of serum miR-92 and miR-122, as well as the LUS score, can predict the short-term disease progression of NRDS.^27^ The combined use of miR-1246 and LUS has the potential to enhance the accuracy of predicting the prognosis of patients with NRDS.^28^ The association between miR-125b-5p expression and LUS score is still unclear. The correlation between miR-125b-5p expression and LUS score was analyzed, and a significant positive correlation was observed, suggesting that increased miR-125b-5p levels may be related to the aggravation of lung lesions in patients. Numerous studies have confirmed that the predictive performance of combined multi-indicator models is significantly superior to that of a single biomarker. For instance, Liang et al. reported that the combination of miR-221-3p, arterial blood gas parameters, and the LUS score for predicting the prognosis of NRDS was significantly superior to that of any single indicator.^29^ Du et al. also found, through logistic regression analysis, that miR-513a-3p may be a risk factor for poor prognosis in NRDS.^30^ Consistent with the above findings, the combined multi-indicator analysis of the present study revealed that after constructing a combined prediction model using the expression level of miR-125b-5p, the LUS score, and umbilical artery blood gas parameters, the ability to distinguish long-term pulmonary adverse events in NRDS patients was significantly enhanced, suggesting that miR-125b-5p can serve as an independent long-term prognostic risk factor.

This study has some limitations. Firstly, the prognostic indicators were based on follow-up results at a corrected age of 12 months, which differs from the traditional approach for short-term prognosis assessment of NRDS. Long-term follow-up may be affected by confounding factors such as post-discharge care and rehabilitation, as well as loss to follow-up. In subsequent studies, short-term prognostic indicators during the first hospitalization will be used for such assessment. Secondly, the sample for this study was collected retrospectively, only including children whose medical records were complete and who had completed the follow-up. Cases of transfer, loss to follow-up, and incomplete data were excluded, which may lead to the omission of high-risk and critically ill children and underestimate the risk of adverse outcomes. Finally, this was a single-center retrospective study, with center-specificities in the enrolled patients and treatment plans, resulting in poor external validity. Additionally, the sample size was limited, and the multi-factor model had the risk of overfitting. There was no external validation cohort, and the stability of the conclusion was insufficient. In the future, a prospective study will be conducted to standardize data collection, actively track lost and transferred cases, reduce selection bias and information loss, and optimize the control of confounding factors.

In conclusion, the present study found that the expression of miR-125b-5p in umbilical cord blood was significantly correlated with the lung ultrasound score and arterial blood gas parameters in NRDS. Its combination with the LUS score and blood gas parameters can significantly improve the prognostic predictive performance. miR-125b-5p is expected to become a novel non-invasive biomarker for the assessment and prognostic stratification of NRDS.

## Data Availability

The dataset that originated the article is available with the corresponding author.
